# Internet non-use among Canadian indigenous older adults: Aboriginal Peoples Survey (APS)

**DOI:** 10.1186/s12889-020-09659-5

**Published:** 2020-10-15

**Authors:** Hossam Ali-Hassan, Rama Eloulabi, Asvini Keethakumar

**Affiliations:** 1 Glendon College, York University, Toronto, ON Canada; 2 Faculty of Health, York University, Toronto, ON Canada

**Keywords:** Internet non-use, Indigenous older adults, Canada, Aboriginal peoples survey

## Abstract

**Background:**

Older adults benefit considerably from Internet use, as it can improve their overall health and quality of life, for example through accessing healthcare services and reducing social isolation. The aim of this study is to assess the prevalence and characteristics of Indigenous older adults in Canada who do not use the Internet.

**Methods:**

The Aboriginal Peoples Survey (APS) 2017 was used and analysis was restricted to those above 65 years of age. The main outcome variable was non-use of the internet in a typical month. Multivariable logistic regression was conducted to assess the relationship between each of the sociodemographic, socioeconomic, lifestyle and health factors and internet non-use.

**Results:**

The prevalence of Indigenous older adults who reported never using the Internet in a typical month was 33.6% with the highest prevalence reported by residents of the Canadian territories while the lowest prevalence was reported in British Columbia. After adjustment, results indicated that older age (OR = 4.02, 95% CI 3.54–4.57 comparing 80+ to 65–69 years of age), being a male (OR = 1.52, 95% CI 1.41–1.63), married (OR = 1.34, 95% CI 1.25–1.44), and living in rural areas (OR = 1.95, 95% CI 1.79–2.13) increased the odds of not using the Internet. First Nation individuals and those who have a strong sense of belonging to the Indigenous identity were more likely to not use the Internet compared to their counterparts. In addition, those who were less educated (OR = 8.74, 95% CI 7.03–1 0.87 comparing less than secondary education to Bachelor’s Degree and above), unemployed (OR = 1.41, 95% CI 1.26–1.57), smoked cigarettes, used marijuana and those with lower self-perceived mental health and unmet health needs were at increased odds of Internet non-use compared to their counterparts.

**Conclusions:**

Findings from this study show that a large proportion of the Indigenous older adults in Canada do not use the internet. It is necessary to address Indigenous communities’ lack of internet access and to create interventions that are consistent with Indigenous values, traditions, and goals.

## Background

Older adults, ages 65 years or older, benefit considerably from Internet use, as it can improve their overall health and quality of life. Homebound or disabled older adults may use the Internet to search for health information, participate in telehealth interventions, support groups and self-management programs, and communicate with health-care professionals online [1].

Many health care providers suggest online health services can be used to improve patient-provider communication, in the areas of patient care and education, and compliance with treatment regimens [2]. Moreover, Internet use among older adults is associated with reduced ageing-related health literacy decline [3]. Low health literacy is associated with low use of self-care and preventative services, excessive use of emergency care services, and increased risk of mortality [3]. Furthermore, Internet access allows older adults to take advantage of online banking, shopping, bill paying, and appointment making [1] and is essential during pandemics and other periods of mandated physical isolation. Older adults, particularly those from disadvantaged communities, can also access resources and services online otherwise unavailable to them. For example, Indigenous women take advantage of online entrepreneurship opportunities to feature their artwork and craftsmanship [4].

Internet use has other benefits, including prevention of social isolation. Social isolation and exclusion are associated with emotional distress, loneliness, depression, poor overall physical and mental health, and reduced quality of life for seniors [5]. Over 30% of Canadians in late adulthood are at risk of becoming socially isolated [5]. By accessing services online rather than relocating closer to providers, older adults from minority or remote communities can avoid the increased risk of social isolation caused by the separation from their social and cultural support at home [6]. Alternatively, older adults may create new, or maintain existing, connections with those not geographically close through online communication. Having social support and good social relations has been linked to better health and a longer life [6]. Erickson and Johnson (2011) [7] also found significant positive links between the use of information communication technologies and the well-being of Canadian elderly. Well-being includes life satisfaction, self-efficacy and social support [7]. According to Ford and Ford (2005), Internet use reduces depressive symptoms in older adults, with an estimated 20% reduction (or more) in the probability of depression classification [8].

Despite these benefits, older adults are less likely to be online than younger adults, a well-documented trend in Western countries [1, 9–12]. Middleton and Sorenson (2006) [10] write that although less than 30% of Canadian households were not using the Internet in 2003, more than 70% of households headed by an older adult did not. More recently, Ali-Hassan, Sekharan and Kim (2019) [13] reported that in Canada 31.9% of older adults did not use the Internet in the last month. Older adults are a vulnerable population with limited internet use due to numerous health concerns and medical conditions and lack of access to technology [2]. Older adults might not have sufficient time or funds, have low social and psychological capital, or suffer from age-related functional restrictions, which decreases their likelihood of being online [9, 11, 12]. Other perceived barriers to Internet access are a lack of Internet attitude, experience and skills, “feeling too old,” or a fear of technology, exacerbated by rapid hardware and software development [11, 12]. In the study conducted by Ali-Hassan, Sekharan and Kim (2019) [13] results showed that education, social class standing, general health, and mental health were some characteristics associated with Internet non-use among Canada’s older adult population [13].

Internet non-use rates are even higher for Indigenous older adults, at 42.4%, in comparison to their non-Indigenous counterparts in Canada [13]. This difference primarily stems from economic and geographical barriers. A digital divide exists for many rural, remote, northern and Indigenous communities as telecommunications and digital services for these communities are unaffordable and inadequate [14]. The cost is too high to get connected, whereas cheaper slow-connection packages do not allow for full use of the Internet and access to all its benefits [4]. Indigenous older adults can especially benefit from the Internet as they are a particularly vulnerable group. Compared to non-Indigenous Canadian older adults, a significantly larger proportion of First Nations, Inuit and Metis older adults live in poverty and suffer from poor health with multiple chronic conditions and disabilities [15]. Similarly, Wilson, Rosenburg, Abonyi, and Lovelace (2010) [16] found significantly more Indigenous seniors in Canada reported fair/poor health and activity limitation than did non-Indigenous seniors. In addition, Indigenous older individuals are at high risk of experiencing social isolation due to consistent oppression, racism, marginalization of language and culture, and historic negative experiences [6]. The identity denial of Indigenous people “through a systematic and concerted effort to extinguish their culture, language and spirit” as well as the intergenerational trauma they experience has led to an estimated 19 to 24% of all Indigenous seniors being socially isolated [6].

Despite the projection that the proportion of Indigenous individuals aged 65 and older will more than double by 2036 [6], it seems little research has been published to address their Internet non-use and lack of access to information technology. Indigenous older individuals in Canada have unique experiences and needs, that too often make them unable to use and uninterested in using the Internet. By studying the characteristics of non-users, as well as the motives behind use and non-use, we enable the development of targeted interventions and policies, and can inform innovators of technology on how to ensure older adults are able to reap the many benefits available online [17]. For example, the report, “Social Isolation of Seniors - A Focus on Indigenous Seniors in Canada” (2018) [6], suggests Indigenous older individuals can be protected against social isolation by “having translators when needed” and having adequate social contact and social support, all of which can be found online. Thus, the aim of this study is to use the data from the 2017 Aboriginal Peoples Survey (APS) [18] to assess the prevalence and characteristics of Indigenous older adults in Canada who do not use the Internet.

## Methods

### Study design, population and sample

Analysis for the present study utilized the Aboriginal Peoples Survey (APS) 2017 [18]. The APS is a cross-sectional survey which examines factors such as health, language, housing and mobility among Indigenous peoples. The target population for this study consists of First Nations people living off reserves, as well as Métis and Inuit people across Canada. The APS is a federally funded survey, conducted by Statistics Canada, and supported by the departments of Human Resources and Skills Development Canada, Aboriginal Affairs and Northern Development Canada, and Health Canada. The data collection for this survey began on January 16 and concluded on August 15, 2017. The APS sample was selected from those who identified with being at least one of the following: First Nations, Métis or Inuit; or those who reported being a Status Indian; or a member of a First Nation or Indian band. This survey samples from a large range of individuals ranging from the ages 15 years and older, excluding those who reside in Indigenous settlements and in remote communities within the Canadian Territories. The survey consisted of a 45-min computer assisted interview in person, by phone or a combination of both. A more detailed description of the APS design and sampling procedure can be found on the Statistics Canada Website [18]. For the purpose of this study, the survey responses were limited to include all adults who were aged 65 and above at the time of response.

### Main outcome

The main outcome variable, non-use of the internet, was determined by the question: “How often do you use the Internet in a typical month?”. For this question, participants were given the potential choices: “daily”, “a few times a week”, “once a week”, “at least once a month”, “less than once a month”, and “never”. The question was further dichotomized into “internet non-use” (for those who answered “never” to the original question) and “internet use” (for those who answered with “daily”, “a few times a week”, “once a week”, “at least once a month”, “less than once a month” to the original question).

### Covariates

A wide range of variables were considered for this analysis. These covariates included sociodemographic factors, socioeconomic factors, lifestyle factors as well as health factors. The sociodemographic factors consisted of age (65–69, 70–74, 75–79, 80+), Indigenous identity (Metis, First Nations, Inuit, Multiple Indigenous Identities), sex (female, male), province/territory of residence (British Columbia, Alberta, Saskatchewan, Manitoba, Ontario, Québec, Eastern Atlantic (including Newfoundland & Labrador, Nova Scotia, Prince Edward Island and New Brunswick), Territories (including Yukon, Northwest Territories & Nunavut)), population center (Rural: less than 1000 residents; Smaller Urban: 1000–99,999 residents; Large Urban: 100,000+ residents), marital status (Single/Not Married, Married/Common Law), mother tongue (French, English, Indigenous Language, Other), residential school experience defined as either Individual and/or Familial attendance (yes, no, not stated), and sense of belonging to own indigenous community measured as having a deep sense of belonging to one’s [First Nations/Métis/Inuit/Aboriginal] group (Strongly Disagree/Disagree, Neither Agree or Disagree, Strongly Agree/Agree).

The socioeconomic variables included education (Bachelor’s Degree and above, Postsecondary below Bachelors, Some-post secondary, Secondary/Some secondary, Less than secondary), employment status (yes, no), yearly income (60,000 + (Highest), 30,000–59,999 (Middle), 5000–29,999 (Lower Middle), Less than 5000 (Lowest)), and residential stability calculated by the number of times moved in a period of 5 years (0 Times (Stable), 1–3 Times (Moderately Stable), 4+ Times (Unstable)).

Lifestyle factors included past-year alcohol use (Regular, Occasional, Non-drinker), current cigarette smoking (yes, no), past-year cannabis use (yes, no), illicit drug use defined as the use of prescription drugs for recreational purposes or use of street drugs including cocaine, speed, solvents, and steroids (yes, no), and access to basic needs captured by the question “Overall, in the past 12 months, was your household income enough to meet your household’s needs for transportation, housing, food, clothing and other necessary expenses? Was it...? ” with answers provided being (More than enough, enough and, not enough).

Finally, variables for health factors consisted of self-perceived general health (Very Good/Excellent, Good, Fair/Poor), self-perceived mental health (Very Good/Excellent, Good, Fair/Poor), access to regular medical doctor (yes, no), and unmet health needs (yes, no).

### Statistical analysis

Bivariate associations between the independent variables and the outcome, Internet non-use, were conducted. Additionally, multivariable logistic regression was conducted to adjust for all the covariates, including sociodemographic, socioeconomic, lifestyle and health factors. Subsequently, Odds Ratios (ORs) and 95% Confidence Intervals (CIs) were reported for both unadjusted and adjusted analysis. Population weights were applied to each calculated estimate and bootstrapping was performed to adjust for the complex sampling methodology. Statistical significance was set at an alpha of 0.05. All analyses were conducted using The Statistical Package for Social Science (SPSS), version 24.0 [19] and Stata Statistical Software, version 13 [20].

## Results

**Fig. 1:**
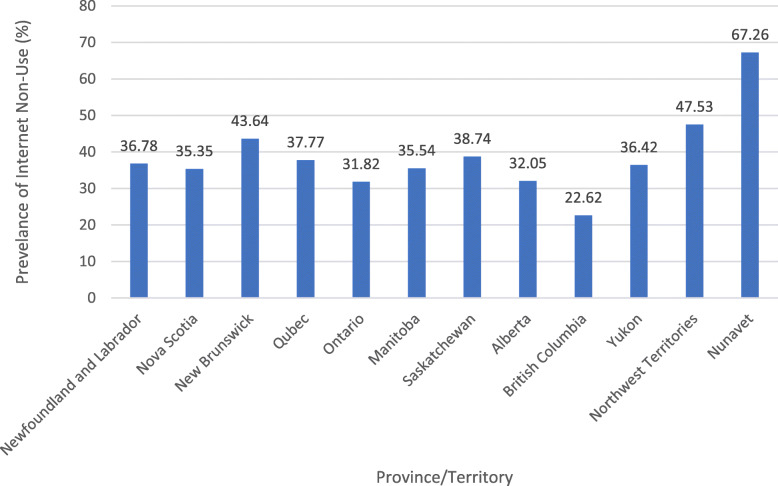
Prevalence of internet non-use among Canadian Indigenous older adults by province and territory (weighted population of 99,140)

The final sample comprised of a weighted population of 99,140 indigenous Canadians aged 65 years and older in the different provinces and territories of Canada based on the Aboriginal Peoples Survey, 2017 [18]. All provinces and territories are reported, with the exception of Prince Edward Islands due to a low sample size. Approximately 33.6% of the respondents reported never using the internet in a typical month. The prevalence of those reporting internet non-use per province/territory is illustrated in Fig. 1. The highest prevalence of using no internet is reported by residents in Nunavut (67.26%), while the lowest prevalence is reported in British Columbia (22.62%).

Results of the unadjusted and adjusted multivariate analysis are displayed in Table 1. The table includes frequencies, unadjusted and adjusted odds ratios (ORs) and 95% confidence intervals (95% CIs) of internet non-use by Indigenous peoples aged 65 years and older living off reserves in Canada, based on the Aboriginal Peoples Survey, 2017 [18].

**Table 1:** Frequencies, unadjusted, and adjusted odds ratios (ORs) and 95% confidence intervals (95% CIs) of internet non-use by older Indigenous Canadians

	N (%)	% Internet non-use	Unadjusted OR (95% CI)	Adjusted OR (95% CI)
**Sociodemographic Factors**				
**Age**				
65–69	44,660 (45.04)	23.64	1	1
70–74	29,560 (29.82)	32.76	**1.57 (1.48–1.68)**	**1.40 (1.29–1.51)**
75–79	14,810 (14.94)	43.60	**2.50 (2.31–2.69)**	**1.68 (1.52–1.86)**
80+	10,110 (10.2)	65.38	**6.10 (5.57–6.67)**	**4.02 (3.54–4.57)**
**Indigenous Identity**				
Metis	50,760 (51.7)	27.65	1	1
First Nations	43,000 (43.79)	39.34	**1.70 (1.61–1.79)**	**1.60 (1.49–1.72)**
Inuit	3220 (3.28)	54.78	**3.17 (2.98–3.37)**	**1.19 (1.05–1.35)**
Multiple Indigenous Identities	*	*	**0.51 (0.39–0.66)**	1.06 (0.80–1.39)
**Sex**				
Female	52,770 (53.23)	32.47	1	1
Male	46,370 (46.77)	34.91	**1.12 (1.06–1.17)**	**1.52 (1.41–1.63)**
**Province**				
British Columbia	14,190 (14.31)	22.62	1	1
Alberta	11,170 (11.26)	32.05	**1.61 (1.47–1.77)**	**1.71 (1.52–1.93)**
Saskatchewan	5960 (6.01)	38.74	**2.16 (1.96–2.39)**	**1.84 (1.61–2.11)**
Manitoba	9380 (9.46)	35.54	**1.89 (1.71–2.08)**	**1.76 (1.53–2.02)**
Ontario	26,500 (26.73)	31.82	**1.60 (1.46–1.74)**	**1.32 (1.18–1.48)**
Québec	16,930 (17.07)	37.77	**2.08 (1.89–2.29)**	**2.03 (1.74–2.36)**
Eastern-Atlantic Provinces	12,640 (12.75)	37.53	**2.05 (1.85–2.28)**	**1.22 (1.06--1.40)**
Territories	2370 (2.39)	55.04	**4.19 (3.85–4.55)**	**1.34 (1.18–1.52)**
**Population Centre**				
Large Urban	33,200 (33.48)	25.74	1	1
Smaller Urban	32,200 (32.48)	34.27	**1.50 (1.41–1.60)**	**1.33 (1.22–1.45)**
Rural	33,740 (34.03)	40.73	**1.98 (1.86–2.11)**	**1.95 (1.79–2.13)**
**Marital Status**				
Single/Not Married	58,980 (59.49)	28.53	1	1
Married/Common Law	40,160 (40.51)	41.15	**1.75 (1.66–1.85)**	**1.34 (1.25–1.44)**
**Mother Tongue**				
French	25,180 (25.4)	34.60	1	1
English	64,190 (64.75)	29.97	**0.81 (0.76–0.86)**	1.07 (0.95–1.20)
Indigenous Language	7900 (7.97)	58.13	**2.62 (2.41–2.86)**	**1.38 (1.18–1.63)**
Other	1870 (1.89)	42.84	**1.42 (1.22–1.65)**	**1.33 (1.08–1.63)**
**Residential School Experience**				
No	44,570 (44.95)	30.86	1	1
Yes	26,650 (26.88)	38.35	**1.39 (1.32–1.48)**	1.07 (0.99–1.16)
Not stated	27,930 (28.17)	33.44	**1.13 (1.06–1.20)**	0.99 (0.91–1.08)
**Belonging to Indigenous Group**				
Strongly Disagree/Disagree	18,980 (20.49)	22.49	1	1
Neither Agree or Disagree	6660 (7.19)	19.82	**0.85 (0.75–0.96)**	**0.84 (0.71–0.98)**
Strongly Agree/Agree	67,000 (72.32)	35.62	**1.91 (1.77–2.05)**	**1.42 (1.30–1.56)**
**Socioeconomic Factors**				
**Education**				
Bachelor’s Degree and above	8920 (9.25)	4.52	1	1
Postsecondary below Bachelors	30,980 (32.14)	24.52	**6.86 (5.64–8.33)**	**2.97 (2.40–3.67)**
Some-post secondary	11,740 (12.18)	23.83	**6.60 (5.40–8.09)**	**2.85 (2.28–3.57)**
Secondary/Some secondary	27,170 (28.19)	35.25	**11.50 (9.45–13.98)**	**4.99 (4.04–6.16)**
Less than secondary	17,580 (18.24)	67.04	**42.94 (35.25–52.31)**	**8.74 (7.03–10.87)**
**Total Personal Income**				
60,000 + (Highest)	9340 (10.78)	8.58	1	1
30,000–59,999 (Middle)	25,490 (29.43)	20.92	**2.82 (2.46–3.24)**	**1.66 (1.39–1.97)**
5000–29,999 (Low Mid)	49,820 (57.51)	42.05	**7.73 (6.82–8.77)**	**2.47 (2.08–2.92)**
Less than 5000 (Lowest)	1970 (2.28)	49.88	**10.61 (9.79–12.80)**	**3.45 (2.73–4.37)**
**Employment**				
Employed	15,740 (15.88)	16.28	1	1
Unemployed	83,350 (84.12)	36.88	**3.00 (2.77–3.26)**	**1.41 (1.26–1.57)**
**Residential Stability**				
0 Times (Stable)	72,090 (73.52)	33.28	1	1
1–3 Times (Moder. Stable)	23,880 (24.36)	33.77	1.02 (0.96–1.08)	**0.88 (0.82–0.95)**
4+ Times (Unstable)	2080 (2.12)	31.21	0.91 (0.74–1.13)	**1.26 (1.02–1.56)**
**Lifestyle Factors**				
**Alcohol Use**				
Regular	42,810 (43.64)	23.29	1	1
Occasional	16,300 (16.62)	31.53	**1.51 (1.41–1.63)**	**1.26 (1.15–1.38)**
Non-drinker	38,970 (39.73)	45.95	**2.80 (2.65–2.96)**	**1.59 (1.48–1.72)**
**Smoking**				
No	80,840 (82.04)	31.20	1	1
Yes	17,700 (17.96)	44.71	**1.78 (1.68–1.89)**	**1.73 (1.59–1.87)**
**Marijuana**				
No	89,270 (95.92)	31.68	1	1
Yes	3800 (4.08)	33.08	1.07 (0.93–1.22)	**1.24 (1.05–1.46)**
**Recreational/Street Drugs**				
No	92,190 (99.16)	31.54	1	1
Yes	780 (0.84)	53.92	**2.54 (2.03–3.17)**	**1.70 (1.30–2.22)**
**Basic Needs**				
More than enough	21,240 (21.53)	18.92	1	1
Enough	56,490 (57.28)	33.36	**2.14 (2.00–2.31)**	**1.56 (1.41–1.73)**
Not enough	20,900 (21.19)	49.08	**4.13 (3.80–3.49)**	**2.20 (1.95–2.50)**
**Health Factors**				
**Self-perceived General Health**				
Very Good/Excellent	37,040 (37.44)	23.12	1	1
Good	32,190 (32.54)	36.41	**1.90 (1.79–2.03)**	**1.23 (1.13–1.35)**
Fair/Poor	29,690 (30.02)	43.63	**2.57 (2.42–2.74)**	**1.10 (1.00–1.21)**
**Self-perceived Mental Health**				
Very Good/Excellent	56,300 (60.09)	24.59	1	1
Good	28,830 (30.76)	42.79	**2.29 (2.16–3.43)**	**1.41 (1.31–1.53)**
Fair/Poor	8580 (9.15)	41.52	**2.18 (2.00–2.37)**	**1.43 (1.27–1.61)**
**Regular Medical Doctor**				
Yes	92,020 (93.48)	32.67	1	1
No	6420 (6.52)	47.12	**1.84 (1.69–1.99)**	**1.31 (1.17–1.48)**
**Unmet Health Needs**				
Yes	9500 (9.67)	31.10	1	1
No	88,720 (90.33)	33.84	**1.13 (1.04–1.24)**	**1.45 (1.30–1.60)**

*indicates values that are not disclosed due to small sample size

Results in bold are significant at alpha of 0.05

At the multivariate level, among the sociodemographic factors, the older the participants, the more likely they were to not use internet. Additionally, those who claim First Nations (OR: 1.60, 95% CI: 1.49–1.72) heritage, were at increased odds of not using internet, as compared to those who claim to be Metis. When examining other sociodemographic characteristics, it is noted that older indigenous males were 1.52 times more likely to not use the internet, compared to indigenous females (95% CI: 1.41–1.63). Among place of residence, all of the Canadian provinces and the territories were at an increased odd of not using the internet, when compared to British Columbia. Additionally, those living in rural (OR: 1.95, 95% CI: 1.79–2.13) residences were significantly more likely not to use internet, compared to their counterparts who resided in larger urban centers. When examining socioeconomic factors, education and income were both clear indicators of internet non-use, as Table 1 illustrates, the lower the education or income level, the more likely individuals were to report internet non-use. Additionally, unemployment was a statistically significant predictor of internet non-use (OR: 1.41, 95% CI: 1.26–1.57), along with residential instability (OR: 1.26, 95% CI: 1.02–1.56). Contrastingly, those who report having moderate stability, and moving residences 1–3 times over the course of 5 years, were less likely to report internet non-use, compared to those being residentially stable (OR: 0.88, 95% CI: 0.82–0.95). Among lifestyle factors, current smokers (OR: 1.73, 95% CI: 1.59–1.87), past 12-month marijuana users (OR: 1.24, 95% CI: 1.05–1.46), and recreational and street drug users (although the prevalence was low at 0.8%) (OR: 1.70, 95% CI: 1.30–2.22), were more likely to not use the internet compared to their non-substance using counterparts. On the other hand, occasional drinkers were (OR: 1.59, 95% CI: 1.48–1.72) more likely to report internet non-use compared to their regular alcohol using counterparts. Additionally, those that report not having enough to access their basic needs (OR: 2.20, 95% CI: 1.95–2.50) or just having enough for their basic needs (1.56, 95% CI: 1.41–1.73), were more likely to report non-use of internet compared to those that report having more than enough to access their basic needs. Lastly when examining health factors, those that reported good (OR: 1.23, 95% CI: 1.13–1.35) general health were more likely to not use the internet compared to those who had very good/excellent general health. Similarly, fair/poor (OR: 1.43, 95% CI: 1.27–1.61) and good (OR: 1.41, 95% CI: 1.31–1.53) mental health were more likely to not use the internet, compared to those with very good/excellent mental health. Individuals without a regular medical doctor (OR: 1.31, 95% CI: 1.17–1.48) and those with unmet health needs (OR: 1.45, 95% CI: 1.30–1.60) were both at an increased odds of not using the internet, compared to their counterparts.

## Discussion

This study aimed to examine the prevalence and characteristics of Indigenous adults, ages 65 and above, who reported never using the Internet in a typical month. Overall, 33.6% of Indigenous older adults living off-reserves in Canada did not use the Internet. After adjustment, results indicated those who were older, First Nations individuals, male, lived in Quebec and in rural areas, were married, and had an Indigenous language mother tongue and a strong sense of belonging to their Indigenous identity were more likely to not use the Internet compared to their counterparts. In addition, Indigenous older individuals who were less educated, unemployed, non-drinkers, smokers, had lower personal income, smoked cigarettes, used marijuana and had lower self-perceived mental health and unmet health needs were at increased odds of Internet non-use compared to their counterparts. These findings are significant as Indigenous older adults are clearly not a homogenous group. Those possessing these traits are at a disadvantage because they cannot benefit from the social, educational, and health-related resources available online.

Our analyses suggest that as Indigenous older adults aged, they were more likely to not use the Internet. Around 65% of Indigenous older adults aged 80 years or older reported not using the Internet in the past month, whereas almost 44% of those aged 75–79, almost 33% of those aged 70–74, and only around 23% of those aged 65–69 reported non-use. This finding maybe explained by lack of access to technology and increased medical conditions and age-related functional restrictions which may decrease the likelihood of being online [2, 9, 11, 12]. Such results align with Ali-Hassan et al.’s (2019) [13] findings that Canadian adults aged 70 or older were at increased odds of Internet non-use compared to those ages 65–69. Similar trends were found in studies from the Netherlands [12] and the United States [1, 21]. Moreover, First Nations older adults living off-reserves were at increased odds of not using the Internet compared to Métis older adults. Indigenous communities are not a homogenous population and future research must focus on their distinct needs, to allow tailored intervention. Results indicated that sex was significantly associated with Internet non-use, as older Indigenous males were almost 1.5 times as likely as females to not have used the Internet. On the other hand, Ali-Hassan et al. (2019) [13] found no significant sex differences, and other relevant literature was inconsistent [1, 9, 12, 22].

Geography plays a role in Indigenous populations’ access to the Internet. Before adjustment, Indigenous older adults residing in the Territories, defined as Yukon, Northwest Territories, and Nunavut, had the highest odds of not having used the Internet in the past month. However, after adjusting for other variables, those in Quebec, Saskatchewan, Manitoba, and Alberta were at increased odds of Internet non-use compared to their counterparts in British Columbia. Additionally, Indigenous older adults living in rural areas were significantly more likely to have not used the Internet compared to their urban counterparts. Haight, Quan-Haase, and Corbett (2014) [22] reported that Canadians living in urban areas were 51% more likely to access the Internet compared to those in rural areas. Off-reserve Indigenous populations are more likely to live in rural areas than their non-Indigenous counterparts [23]. A strong digital divide exists for Northern and remote communities versus other Canadian communities [4]. Internet connection in remote rural areas is slow, unreliable, and expensive [23]. In Quebec for example, most remote Indigenous communities access the Internet by satellite and have a maximum speed of less than 1.5 megabits per second, compared to a basic speed of 30 megabits per second in Montreal [24]. This form of Internet frequently disconnects, making video-conferencing and downloading files almost impossible [24].

Our analyses indicated that married or common law Indigenous older adults were at increased odds of not having used the Internet compared to their single counterparts. Previous literature showed similar associations between marital status and Internet use [25]. Ali-Hassan et al. (2019) [13] further reported that those who had partners or significant others and those who were satisfied with their personally relationships were more likely to not have used the Internet. Therefore, it is probable that married or common-law Indigenous older adults are not motivated to use the Internet as they do not feel the need to connect with others or socialize online. Moreover, Indigenous older individuals whose mother tongue was an Indigenous language were more likely to not have used the Internet than those whose mother tongue was French. Individuals who reported having a deep sense of belonging to their Indigenous identity were also at increased odds of Internet non-use compared to those who did not. These results suggest a digital exclusion, potentially tied to the limited online resources available in Indigenous languages, lack of culturally appropriate content online and potential exposure to racism, made bolder by the protection of anonymity [4]. During their interviews with Smillie-Adjarkwa (2005) [4], Indigenous women in Canada mentioned that some sites online do promote cultural, religious, and language racism, despite claims that they did not face racism online. There is no previous literature that investigates the potential relationships that may exist between these variables, but further research is warranted.

In terms of socioeconomic factors, education, income, and employment were associated with Internet non-use. Results demonstrated that the lower the formal education an Indigenous older adult had received, the more likely they were to not use the Internet; those who had not obtained a secondary education were more than 8 times more likely to not use the Internet compared to those who received a Bachelor’s degree or higher. Moreover, Indigenous older adults with lower personal incomes were at increased odds of Internet non-use compared to those with higher personal incomes. Our findings are in line with Ali-Hassan et al. ‘s (2019) [13] results on Canadian older adults and with other studies’ results on older adults in the United States and the United Kingdom [25–27]. Our analysis found that unemployed Indigenous older individuals were at increased odds of Internet non-used compared to employed individuals. Previous literature indicates similar trends between socioeconomic factors and Internet use, identifying cost as a significant barrier to Internet access [13, 25, 26]. These differences in socioeconomic status between users and non-users highlight and often exacerbate the existing social inequalities that further marginalize Indigenous individuals.

Interestingly, results indicated that non-drinkers were at increased odds of Internet non-use compared to alcohol drinkers. Similarly, Canadian older adults who had not consumed alcohol in the past month were more likely to not have used the Internet compared to those who had consumed alcohol [13]. On the other hand, smokers, marijuana users, and users of recreational or street drugs were all at increased odds of not using the Internet compared to their counterparts. Although the relationship between Internet use and these lifestyle factors has been explored in younger populations [28–30], more research is needed on older adults. Furthermore, Indigenous older adults who reported having fair/poor self-perceived mental health were at increased odds of Internet non-use compared to those who reported very good/excellent mental health. Ali-Hassan et al. (2019) [13] found similar results and highlight that although the association is prominent in the literature [31], it is unclear whether mental health plays a causative role in Internet use or the other way around. Internet use may lead to increased autonomy, increased levels of empowerment [32], lower levels of social isolation, which causes overall better mental health of Indigenous older adults. Alternatively, higher feelings of self-efficacy in older adults may be driving them to adopt new skills and use the Internet [13]. It may be that those who have better mental health are more willing to learn new skills such as information and communication technology (ICT) skills or have more self-esteem to get out of their comfort zones. Finally, Indigenous older adults who did not have unmet health needs were more likely to have not used the Internet. It is possible that those with unmet health needs turn to the Internet for information and resources to help address their needs.

### Strengths and limitations

The present study has many strengths. It is the first of its kind, to our knowledge, to examine Internet non-use in Indigenous older adults by analyzing such a comprehensive list of factors. The data has a representative scope, as all Canadian provinces were considered, and therefore allows for better generalizability. Additionally, the relatively large sample size for the APS allowed ample statistical power to draw meaningful conclusions. Nonetheless, the results must be interpreted with care as limitations do exist. Given that the APS data was self-reported, there is potential for information bias. Another major limitation to the study’s generalizability is the exclusion of Indigenous populations living on reserves, as well as those who are transitioning between households or who are homeless. Thus, findings cannot be generalized to all Indigenous populations. Lastly, causal relationships cannot be inferred from our findings due to the nature of the study.

## Conclusions

The Internet has the potential to be reduce obstacles and issues faced by Indigenous older adults, such as social isolation. Our findings shed some light on the digital divide that exists for Indigenous communities and highlight existing disparities. It is necessary to address Indigenous communities’ lack of internet access and to create interventions that are consistent with Indigenous values, traditions, and goals [24]. More in depth qualitative and quantitative studies should aim to understand the needs of the diverse older adult population in Canada and their motivations to use the Internet. Such information may be used to inform and direct services and policies ensuring older adults, especially from Indigenous communities, can reap the Internet’s benefits.

## Data Availability

The data analyzed during the current study is part of Statistics Canada’s 2017 Aboriginal Peoples Survey (APS) dataset. Details about the survey and dataset can be found at www23.statcan.gc.ca/imdb/p2SV.pl?Function=getSurvey&Id=318572 The dataset is accessible at one of Statistics Canada’s Research Data Centres (RDCs). Access to the data requires an application available at www.statcan.gc.ca/eng/microdata/data-centres/access

## Abbreviation

APS: Aboriginal Peoples Survey
